# A Role for Fibrillar Collagen Deposition and the Collagen Internalization Receptor Endo180 in Glioma Invasion

**DOI:** 10.1371/journal.pone.0009808

**Published:** 2010-03-22

**Authors:** Ivo J. Huijbers, Marjan Iravani, Sergey Popov, David Robertson, Safa Al-Sarraj, Chris Jones, Clare M. Isacke

**Affiliations:** 1 Breakthrough Breast Cancer Research Centre, The Institute of Cancer Research, London, United Kingdom; 2 Paediatric Oncology, The Institute of Cancer Research/Royal Marsden NHS Trust, Sutton, United Kingdom; 3 Clinical Neuropathology, King's College Hospital, London, United Kingdom; The University of Chicago, United States of America

## Abstract

**Background:**

Glioblastoma multiforme (GBM, WHO grade IV) is the most common and most malignant of astrocytic brain tumors, and is associated with rapid invasion into neighboring tissue. In other tumor types it is well established that such invasion involves a complex interaction between tumor cells and locally produced extracellular matrix. In GBMs, surprisingly little is known about the associated matrix components, in particular the fibrillar proteins such as collagens that are known to play a key role in the invasion of other tumor types.

**Methodology/Principal Findings:**

In this study we have used both the Masson's trichrome staining and a high resolution multiple immunofluorescence labeling method to demonstrate that intratumoral fibrillar collagens are an integral part of the extracellular matrix in a subset of GBMs. Correlated with this collagen deposition we observed high level expression of the collagen-binding receptor Endo180 (CD280) in the tumor cells. Further, interrogation of multiple expression array datasets identified Endo180 as one of the most highly upregulated transcripts in grade IV GBMs compared to grade III gliomas. Using promoter analysis studies we show that this increased expression is, in part, mediated via TGF-β signaling. Functionally, we demonstrate that Endo180 serves as the major collagen internalization receptor in GBM cell lines and provide the first evidence that this activity is critical for the invasion of GBM cells through fibrillar collagen matrices.

**Conclusions/Significance:**

This study demonstrates, for the first time, that fibrillar collagens are extensively deposited in GBMs and that the collagen internalization receptor Endo180 is both highly expressed in these tumors and that it serves to mediate the invasion of tumor cells through collagen-containing matrices. Together these data provide important insights into the mechanism of GBM invasion and identify Endo180 as a potential target to limit matrix turnover by glioma cells and thereby restrict tumor progression.

## Introduction

High-grade gliomas are the most common brain tumors in adults, and are characterized by their treatment-refractory nature and poor clinical outcome. They are classified into four grades as defined by the World Health Organization [1]. Of these, grade IV glioblastoma multiforme (GBM) tumors have the worst prognosis, with median survival of 10–12 months. GBMs are characterized by extensive microvascular proliferations and/or necrosis in addition to the nuclear atypia and mitotic activity seen in grade II and III tumors [1]. Moreover, complete surgical resection of GBM is difficult due to the infiltration of tumor cells into the surrounding brain tissue [2]. This invasive process is characterized by adhesion of the tumor cells to locally produced extracellular matrix (ECM) components, cell locomotion and the ability of invading cells to remodel their local extracellular space [3], [4].

The normal brain ECM has a unique composition consisting mainly of hyaluronan, proteoglycans and tenascin-C and, apart from the basement membrane of the normal brain vasculature, is devoid of rigid protein barriers formed by fibrillar matrix proteins [5]. In gliomas, the development of extensive microvascular proliferations is associated with a large increase in basement membrane components such as laminin, collagen IV and fibronectin. In addition, an increased deposition of tenascin-C, hyaluronan and vitronectin is found associated with the tumors cells. For the latter two components, this is frequently accompanied by enhanced expression of their respective adhesion receptors, CD44 and αvβ3 integrin [6], [7]. In contrast, surprisingly little is known the fibrillar matrix components that are a major ECM component in other tumor types. For example, it has been reported that the most abundant of the fibrillar collagens, collagen I, is absent in GBMs [8], [9]. Here we have re-examined the presence of collagens in gliomas and show using complementary methods that intratumoral fibrillar collagen can be observed in nearly a third of the GBM cases. Furthermore, we correlate this ECM deposition with the expression of the collagen internalization receptor Endo180 and show that this receptor plays a critical role in glioma invasion.

## Results

### Endo180 is highly expressed in GBM

**Figure 1:**
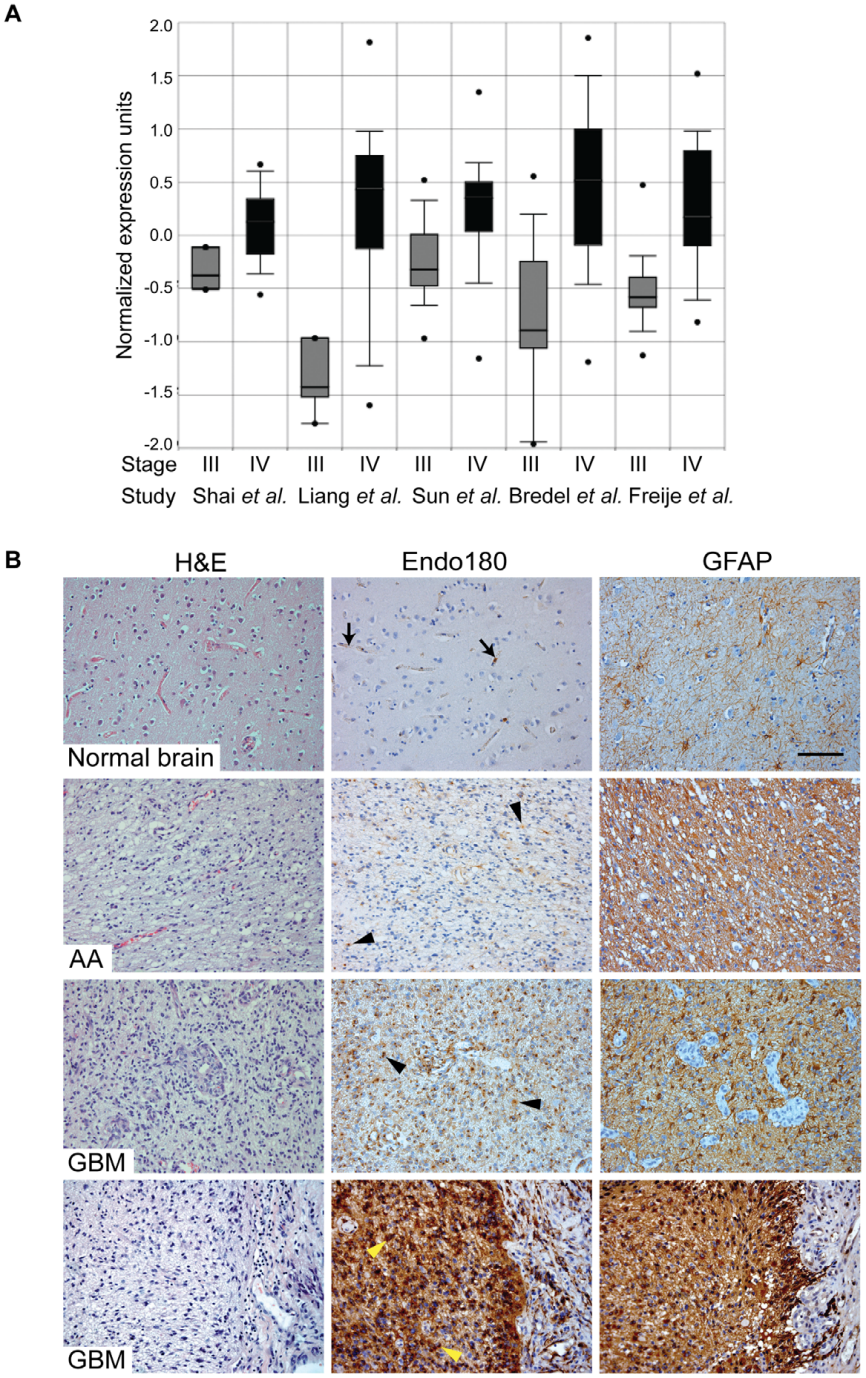
Endo180 expression is highly upregulated in GBMs. (A) Normalized expression of Endo180 in grade III gliomas (astrocytomas, oligodendrogliomas and oligoastrocytomas) and grade IV gliomas (GBMs). Box plots were created by ONCOMINE™ from five independent expression array studies. *p*-values were 5.6×10^−5^ (Shai *et al.*) [22], 1×10^−6^ (Liang *et al.*) [21], 2.2×10^−12^ (Sun *et al.*) [23], 5.9×10^−8^ (Bredel *et al.*) [19] and 2.3×10^−12^ (Freije *et al.*) [20]. (B) FFPE whole tissue sections of normal brain (2 samples) and grade III (2 samples) or grade IV gliomas (9 samples) were H&E stained or immunostained for Endo180 (mAb 39.10) and glial fibrillary acidic protein (GFAP). Representative images of the temporal lobe of normal brain showing weak expression of Endo180 in some cells associated with the vasculature (arrows); a grade III anaplastic astrocytoma (AA) showing weak Endo180 expression in GFAP-positive tumor cells (arrowheads), two grade IV glioblastomas (GBM) showing strong Endo180 expression in tumor cells (black and yellow arrowheads). Scale bar, 100 µm.

Endo180 is large transmembrane glycoprotein that constitutively recycles between the plasma membrane and intracellular endosomes [10], [11], [12]. In normal healthy tissues, expression of Endo180 is predominantly restricted to cells of mesenchymal origin, in particular stromal fibroblasts [13], [14], [15]. Both cell based and *in vivo* experiments have demonstrated that Endo180 binds collagens and functions to internalize them for delivery to, and degradation in, the lysosomes [16], [17], [18]. *In silico* analyses of independent gene expression datasets in ONCOMINE™ [19], [20], [21], [22], [23] revealed that Endo180 (*MRC2*) transcripts were significantly upregulated in grade IV gliomas, i.e. GBMs, versus grade III gliomas (Figure 1A). By combining these five studies, Endo180 was ranked as the 6^th^ most highly upregulated gene in GBMs versus grade III gliomas (p = 5.56×10^−5^) (Table S2). To corroborate the *in silico* analysis, we first examined the expression and distribution of Endo180 protein by immunohistochemistry in 11 archival high-grade glioma samples for which whole tissue sections were available. Representative immunohistochemistry images are shown in Figure 1B. In the 2 grade III anaplastic astrocytomas, 80–90% of the tumor cells were glial fibrillary acidic protein (GFAP) positive and 30–40% of the tumor cells showed low level Endo180 expression. In the 9 GBMs, high level Endo180 expression was detected in 80–100% of tumor cells. The concentration of perinuclear Endo180 staining is consistent with the known distribution of this recycling receptor to intracellular endosomes [11], [12]. Endo180 protein was not detected in the normal brain apart from weak expression in some cells associated with the vasculature.

Next we examined a series of 79 grade III and IV glioma cases collected in a tissue microarray (TMA). We observed Endo180 expression in 62/79 (78.5%) samples. In agreement with the *in silico* transcript analysis, there was a significantly higher proportion of Endo180 positive cases in grade IV GBM (59/69, 85.5%) compared to the grade III lesions anaplastic astrocytoma (1/5, 20%) or anaplastic oligodendroglioma (2/5, 40%) (Table 1, grade IV *versus* grade III, *p* = 0.0005, Fishers exact test).

**Table 1:** Correlation between expression of Endo180 in tumour cells, glioma grade and the presence of intratumoral fibrillar collagen in a high-grade glioma TMA.

		Endo180		
		Negative	Positive	*p*
**Grade**	n = 79			**0.0005**
III		7	3	
IV		10	59	

		Intratumoral Fibrillar Collagen		
		Negative	Positive	
**Grade**	n = 79			0.268
III		9	1	
IV		48	21	

		Endo180		
		Negative	Positive	
**Intratumoral Fibrillar Collagen**	n = 79			**0.026**
Negative		20	37	
Positive		0	22	

79 grade III and IV gliomas arrayed as a TMA were subject to immunohistochemical staining with the anti-Endo180 mAb 39.10. Intratumoral fibrillar collagen was detected using the Masson's trichrome staining method. Clinical pathological details and scoring of the individual tumors is shown in Table S2. Shown here are the statistical correlations calculated using Fisher's exact test. There was a significantly higher proportion of Endo180 positive cases in grade IV versus grade III tumors (*p* = 0.0005). There was no significant difference in the deposition of intratumoral collagen between grade III and grade IV tumors (*p* = 0.268). There was a significant association between intratumoral collagen deposition and Endo180 expression (*p* = 0.026).

**Figure 2:**
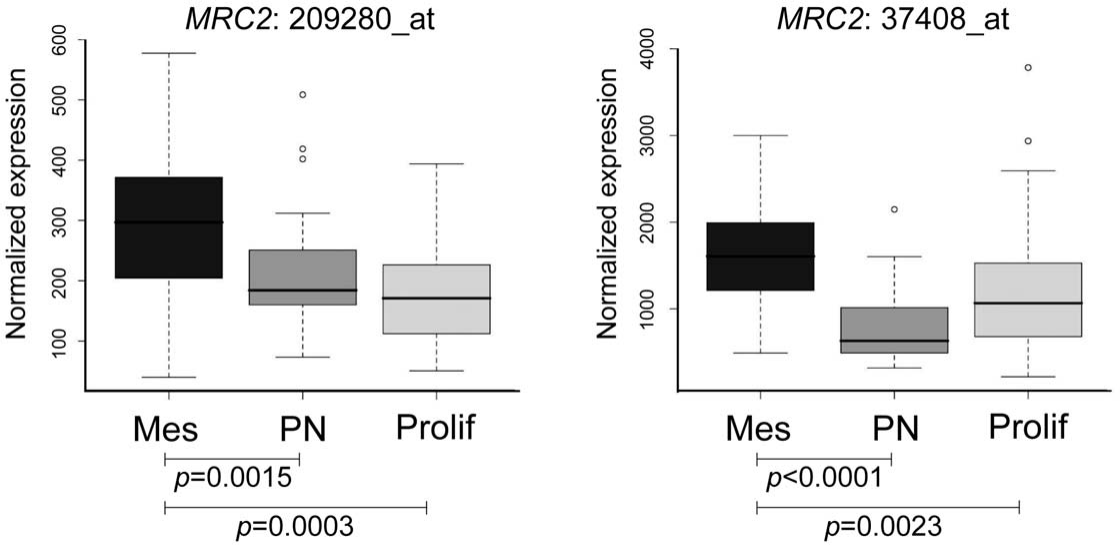
Endo180 expression is associated with the ‘mesenchymal’ subclass of high grade glioma. Boxplots for Endo180 (*MRC2*) expression values in the three molecular subclasses of high-grade glioma described by expression profiling [24]: mesenchymal (Mes), proneural (PN) and proliferative (Prolif). The two *MRC2* probe sets, 209280_at and 37408_at, that were present in the array are significantly associated with the mesenchymal group of tumors (Mann-Whitney U test). Box represents upper and lower quartiles, with median designated by a horizontal line. Whiskers represent the minimum and maximum non-outlier observations, with outliers (open circles) defined as having a value more than 1.5 times the interquartile range lower than the first quartile, or higher than the third quartile.

Recently, Phillips and colleagues have described three prognostic subclasses of high-grade glioma: proneural, proliferative and mesenchymal [24]. Interrogation of their expression profiling data revealed that Endo180 was strongly correlated with the mesenchymal subclass (p<0.005), placing Endo180 positive tumors into a poor survival cohort characterized by increased neovascularization and expression of neural stem cell markers (Figure 2).

### TGF-β regulates Endo180 expression in glioma cell lines

**Figure 3:**
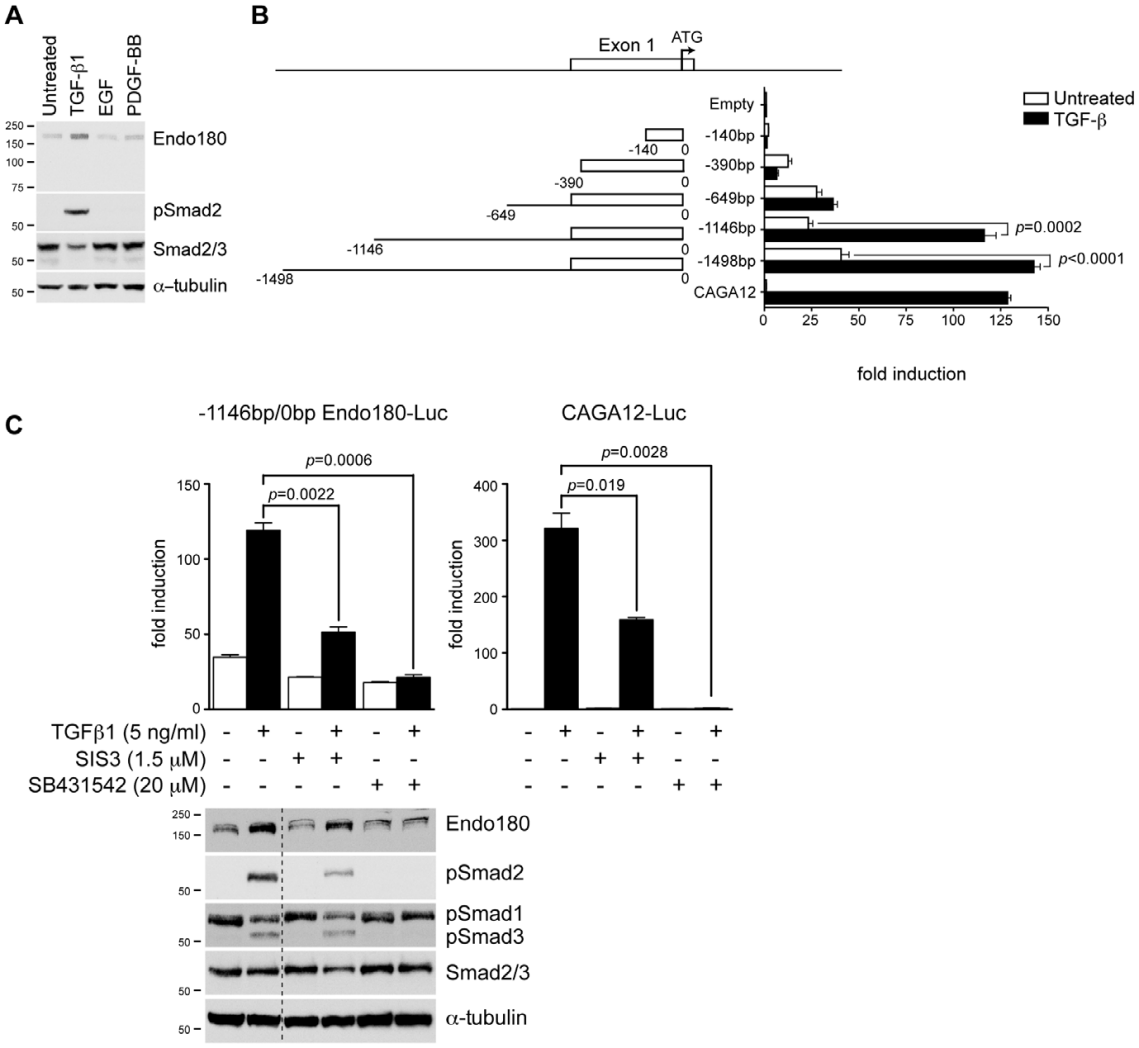
Expression of Endo180 is regulated by TGF-β signaling. (A) U87MG cells were stimulated for 24 h with 5 ng/ml TGF-β1, 50 ng/ml EGF or 50 ng/ml PDGF-BB. Endo180 expression (mAb A5/158) and phosphorylation of the SMAD2 protein was monitored by western blotting. (B) Schematic diagram showing the Endo180 promoter fragments cloned into pGL3-basic. U87MG cells were transfected with Endo180 promoter constructs or control vectors, pGL3-empty and pGL3-CAGA12-Luc, and treated with or without 5 ng/ml TGF-β1 for 24 h. Bars represent mean values from three independent experiments ± SEM. *p*-values were generated using the student's *t*-test. (C) The −1146bp/0bp Endo180 promoter construct or the pGL3-CAGA12-Luc vector were transfected in U87MG cells. Cells were treated with TGF-β1 in the presence or absence of the inhibitors SIS3 or SB431542 for 24 h. Graphs show fold induction as compared to the untreated pGL3-CAGA12-Luc control. Error bars represent two independent experiments performed in triplicate (upper panel). In parallel, activation of TGF-β signaling was monitored by immunoblotting (lower panel). Dotted line indicates lanes taken from the same gel at the same exposure.

Several mechanisms could account for the increased expression of Endo180 in GBMs. We excluded genetic and epigenetic regulation as no genomic amplification of the Endo180 locus (*MRC2*) at 17q23 was detected in 11 glioma cell lines [25] and the Endo180 promoter was unmethylated in all cell lines tested, with the exception of UW479 (Figure S1). Several growth factors pathways, particularly those downstream of the epidermal growth factor (EGF), platelet-derived growth factor (PDGF) and TGF-β receptor families, have been shown to be activated in gliomas [26], [27]. EGF and PDGF-BB treatment of the glioma cell line U87MG had no effect on Endo180 protein levels however TGF-β1 treatment resulted in increased Endo180 expression (Figure 3A). Luciferase reporter assays were performed with human Endo180 promoter fragments to determine the TGF-β responsive region. TGF-β1 treatment resulted in a 5-fold increase in luciferase expression from the −1146bp/0bp Endo180 promoter fragment but only a 1.3-fold increase from the shorter −649bp/0bp fragment (Figure 3B). The −1146bp/0bp Endo180 promoter fragment was then used to determine whether the TGF-β responsiveness was directly mediated via downstream Smad signaling. To address this, U87MG cells were treated with Smad3 inhibitor, SIS3 [28]. The type I TGF-β receptor inhibitor, SB431542, was used as a control for inhibition of total TGF-β signaling [29]. SIS3 treatment caused a significant decrease in Endo180 promoter activity in the presence of TGF-β1 (2.3-fold decrease, *p* = 0.0022) (Figure 3C). This inhibition was similar to the relative decrease observed with the Smad-responsive control vector, CAGA_12_-luciferase (2.0-fold decrease, *p* = 0.0190) [30]. SB431542 treatment completely prevented TGF-β1 induction of Endo180 promoter activity. Immunoblotting confirmed that SB431542 treatment completely prevented the phosphorylation of Smad2 and Smad3, whereas SIS3 treatment was less effective consistent with its partial prevention of Endo180 induction by TGF-β1. Interestingly, both inhibitors reduced the baseline level of Endo180 expression indicating that there is a low-level of autocrine TGF-β stimulation in U87MG cells.

### Collagen uptake in glioma cells is Endo180-dependent

**Figure 4:**
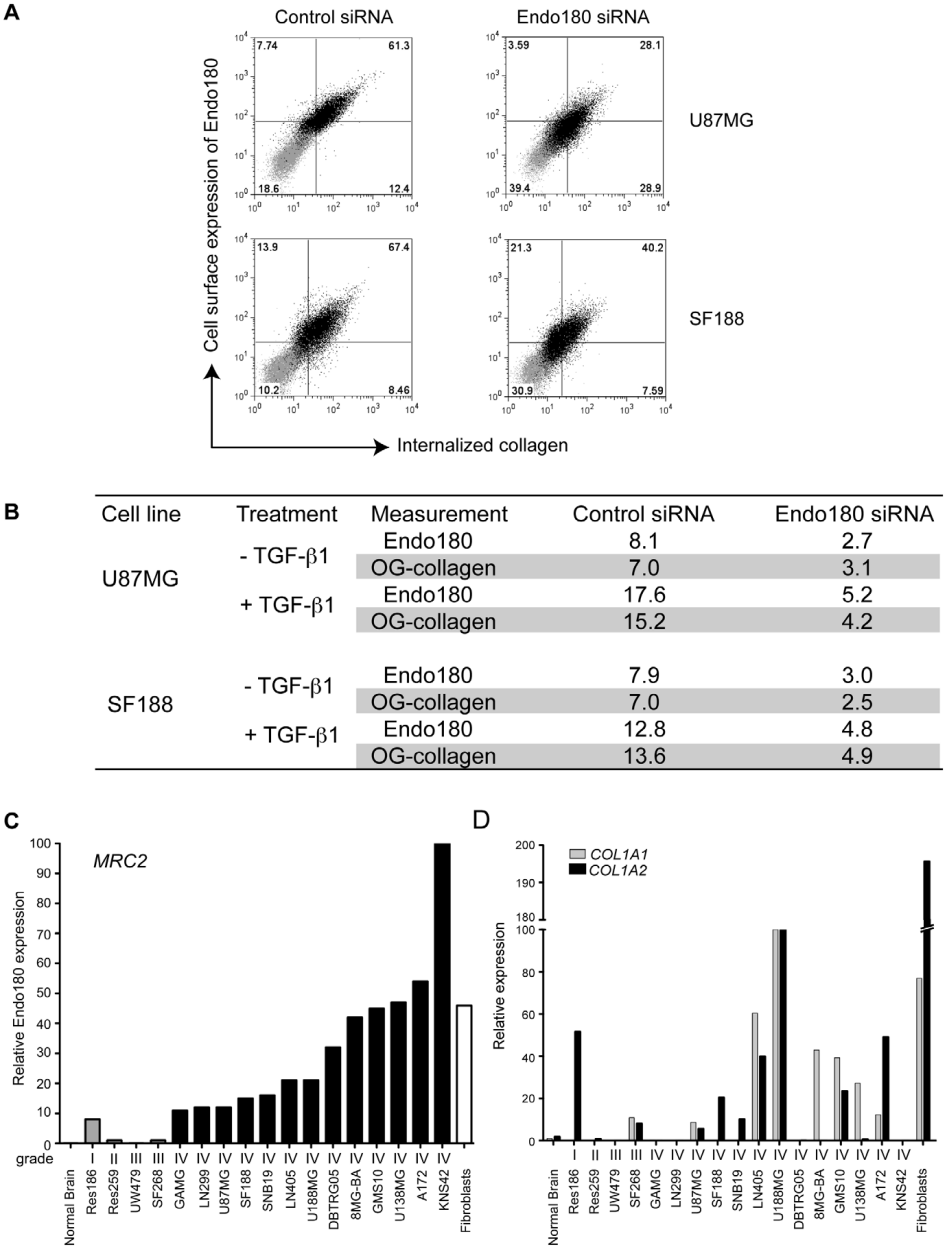
Collagen internalization and expression by glioma cell lines. (A–B) U87MG and SF188 glioma cells were transfected with control or Endo180 siRNA oligonucleotides for 24 h and then cultured for a further 72 h with or without 5 ng/ml TGF-β1. Cells were then cultured for 2 h at 37°C in the presence of 20 µg/ml OG-collagen as previously described [16]. Cells were trypsin treated to remove cell surface associated collagen and the levels of cell surface Endo180 and internalized collagen were monitored by flow cytometry. (A) Dot plots (isotype matched control labeled cells in the absence of OG-collagen in grey, antibody labeled cells in the presence of OG-collagen in black) show that reduced cell surface expression of Endo180 is matched by reduced collagen uptake. (B) Table showing the mean fluorescence intensities normalized to cells stained with a control isotype matched antibody in the absence of OG-collagen. (C–D) Relative expression of Endo180 (*MRC2*), *COL1A1* and *COL1A2* in a panel of 17 glioma cell lines, normal brain and normal human fibroblasts as measured by qPCR. (C) Graph showing relative Endo180 expression related to the highest expresser, KNS42, which was set at 100. All grade IV gliomas (black bars) express higher levels of Endo180 compared to grade III tumors (grey bars). Negligible Endo180 expression was detected in normal brain. (D) Relative expression of the two collagen I genes, *COL1A1* (grey bars) and *COL1A2* (black bars) related to the highest collagen I expresser, U188MG, which was set at 100.

Tumors need to remodel the ECM to physically expand and liberate latent growth factors [31], [32]. A major function of Endo180 is to internalize collagens for intracellular degradation [16], [17], [18]. To test if Endo180 similarly functions as a collagen internalization receptor in glioma cells, U87MG and SF188 cells were treated with Endo180 or control siRNA oligonucleotides and then incubated with OregonGreen (OG)-collagen for 2 h at 37°C. Flow cytometry analysis demonstrated that Endo180 expression was reduced 3.0 and 2.6-fold following Endo180 siRNA treatment in U87MG and SF188, respectively, and that this was matched with a 2.3 and 2.8-fold decrease in collagen uptake (Figure 4A,B). TGF-β1 treatment of U87MG and SF188 glioma lines resulted in a 2.2 and 1.6-fold increase in cell surface expression of Endo180, respectively, and again this was matched with a similar increase in collagen uptake, 2.2 and 1.9-fold (Figure 4B).

### Collagen I is present in the extracellular matrix of GBM

**Figure 5:**
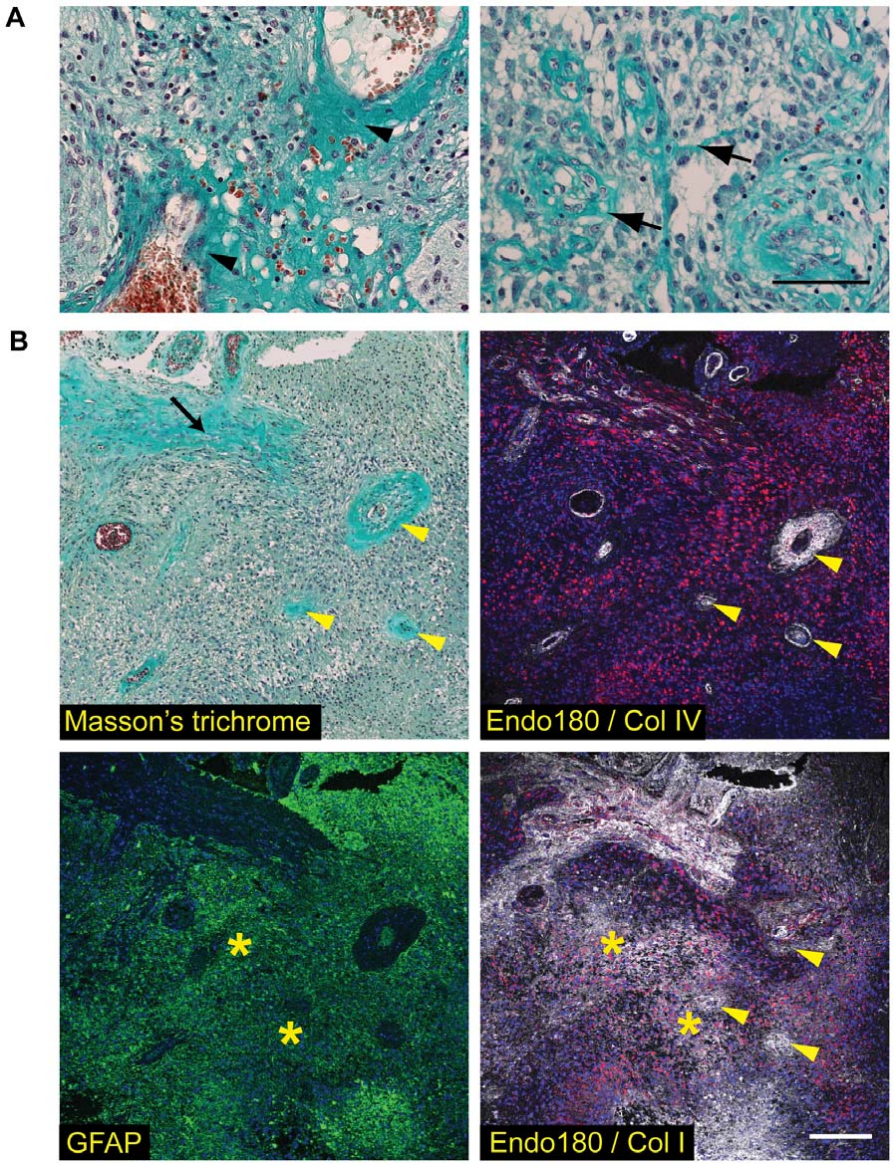
Fibrillar collagens are abundant in the extracellular matrix of GBMs. Staining was performed on GBMs for which whole tissue sections were available. Representative images are shown. (A) GBMs stained with Masson's trichrome. Collagen fibers are stained blue/green, nuclei are black and erythrocytes red. Left hand panel shows extensive collagen deposition in the basement membranes associated with the tumor vessels (arrowheads). Right hand panel illustrates collagen fibers within the tumor mass (arrows). Scale bar, 100 µm. (B) Four adjacent FFPE sections of a GBM were stained as follows: Masson's trichrome to visualize fibrillar collagens (upper left panel), immunolabeled with glial fibrillary acidic protein (GFAP; green) and counterstained with DAPI (blue) to visualize tumor cells (lower left panel), immunolabeled with collagen IV or collagen I (white) and Endo180 (red) and counterstained with DAPI (blue) (upper and lower right panels, respectively). Arrowheads indicate large tumor vessels surrounded by a basement membrane containing mainly collagen IV and some collagen I. Arrow indicates an adjacent stromal region containing dense collagen I fibers and smaller tumor vessels. Asterisks indicate GFAP-positive, Endo180-positive regions with extensive collagen I deposition. Scale bar, 150 µm.

If the highly upregulated collagen internalization receptor Endo180 were to play a functional role in GBMs, it would be anticipated that it would be in intimate contact with a collagen containing extracellular matrix. However, although the basement membrane collagen, collagen IV, is found associated with the extensive microvascular proliferations characteristic of GBMs, it has been reported that the GBMs have low or undetectable levels of the major fibrillar collagen, collagen I [8], [9]. Hence it was important to re-examine whether there is indeed a significant deposition of a fibrillar collagen matrix in GBMs. To address this, a number of approaches were taken. First, interrogation of the ONCOMINE™ microarray data revealed that within the 100 most highly upregulated genes in GBM compared to grade III tumors were fibrillar collagen genes and genes encoding collagen processing enzymes (Table S2). Second, collagen 1 is trimeric protein comprised of two α1 subunits and one α2 subunits encoded by *COL1A1* and *COL1A2* genes, respectively. qPCR analysis of a panel of glioma cell lines with variable Endo180 expression demonstrated substantial expression of both *COL1A1* and *COL1A2* transcripts in a subset of these cell lines as compared to the minor levels detected in normal brain (Figure 4C–D). Of note, *COL1A1*, *COL1A2* and Endo180 (*MRC2*) expression in GBM lines was comparable to that in normal human fibroblasts, which are known to produce high levels of collagen 1 and Endo180 protein. Third, the high-grade glioma samples arrayed in the TMA (Table 1) and available as whole tissue sections (see Figure 1) were also subjected to Masson's trichrome staining to visualize collagen fibers. In all tumor samples, Masson's trichrome staining was detected in association with the angiogenic vasculature resulting from the substantial deposition of basement membrane collagens, in particular collagen IV (Figure 5A–B). Analysis of the tumors in the TMA revealed that 21/69 (30.4%) of GBM samples and 1/10 grade III tumors also showed extensive collagen fibers present within the tumor mass. Interestingly, this tumor-associated collagen staining was significantly associated with Endo180 protein immunopositivity (Table 1, *p* = 0.026, Fishers exact test). Finally, to confirm that the tumor-associated collagen detected by Masson's trichrome staining truly represented the deposition of fibrillar collagens, consecutive formalin-fixed paraffin-embedded (FFPE) glioma whole tissue sections were subject to Masson's trichrome staining and triple immunofluorescent labeling with antibodies against collagen I, collagen IV, Endo180 and GFAP (Figure 5B). Collagen I depositions were observed in three separate areas: intimately associated with high Endo180 expressing, GFAP-positive tumor cells, in GFAP-negative stromal regions, and, to a lesser extent, adjacent to vascular proliferations.

### Endo180 promotes glioma invasion through a collagen matrix

**Figure 6:**
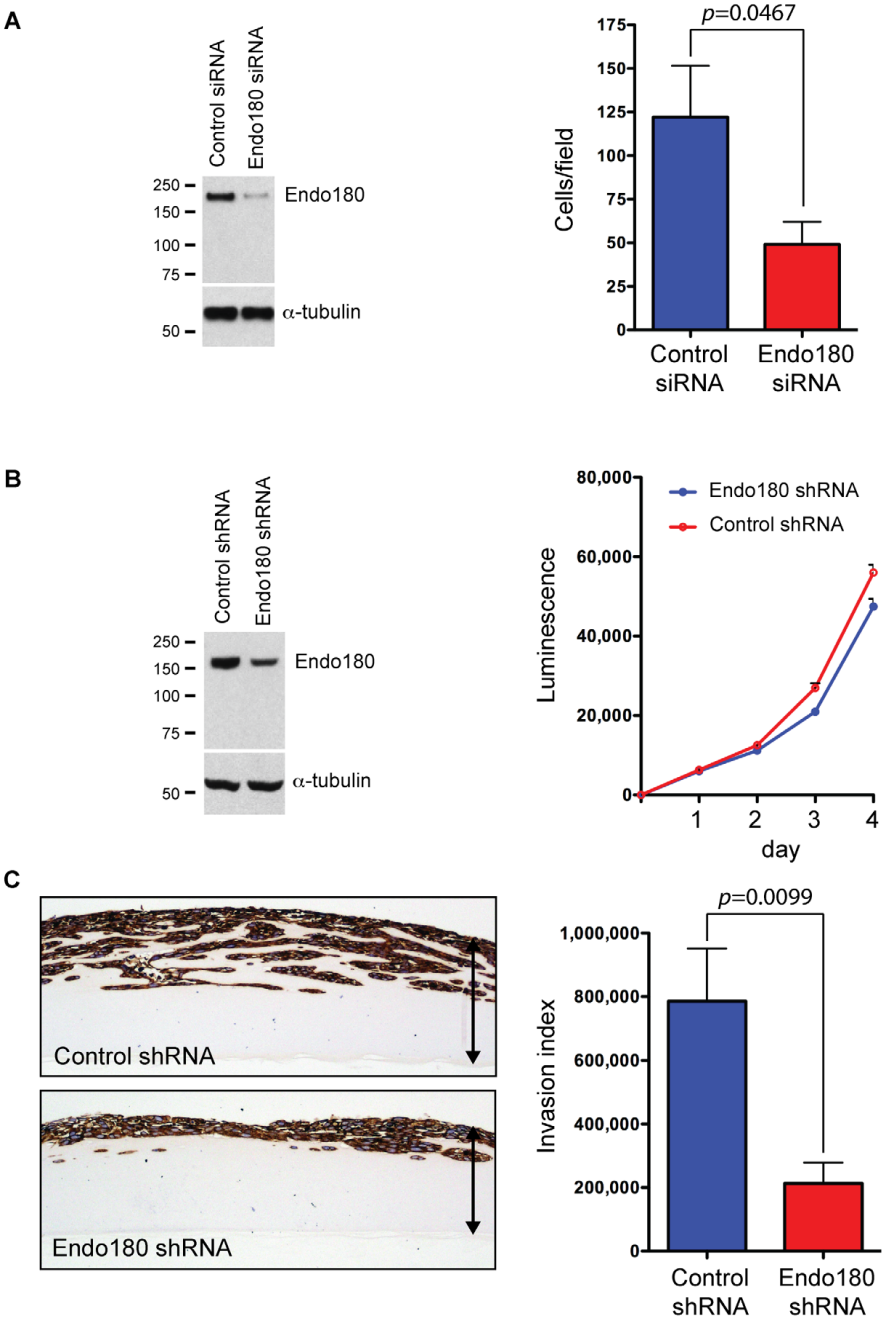
Endo180 mediates invasion of glioblastoma cells through a collagen matrix. (A) SF188 cells were treated with control or Endo180 siRNA oligonucleotides. After 48 h, cells were plated on Transwell inserts coated with collagen I and allowed to migrate towards a high serum concentration for 24 h. Data represents >6 independent experiments. Parallel cultures were subject to immunoblotting to confirm siRNA mediated Endo180 downregulation. (B) Growth of SF188 cells stably infected with control or Endo180 shRNA lentiviruses was monitored over 4 days using Cell Titre-Glo and subject to immunoblotting to confirm shRNA mediated Endo180 downregulation. Error bars represent mean values from two independent experiments each with triplicate samples ±SEM. (C) Control and Endo180 shRNA infected SF188 cells were plated onto a thick collagen I-containing gel and cultured for 11 days. Gels were embedded, sectioned, immunostained with vimentin and an invasion index calculated. Left hand panel shows representative images. Arrows indicate depth of matrix. Right hand panel shows invasion index from 4 independent experiments each with duplicate or triplicate samples. *p*-values were generated using the student's two-tailed *t*-test.

Having demonstrated that a significant proportion of GBMs have fibrillar collagens deposited within the tumor mass, we next addressed whether Endo180 played a functional role in promoting the invasive capacity of these tumors. Despite numerous reports that Endo180 can mediate collagen uptake for lysosomal degradation in a variety of cell types, the role of this receptor in promoting migration through a fibrillar collagen matrix has not been investigated. To address this, we took two complementary approaches. First, we monitored the invasion of the glioma cells lines treated with control or Endo180 siRNA oligonucleotides through non-denatured collagen I coated Transwell filters. Endo180 siRNA treated cells showed a significant inhibition of Transwell migration (59.3% inhibition, *p* = 0.0467) (Figure 6A). Second, cell lines stably infected with lentiviruses containing control or Endo180 shRNAs were generated. Of note, the shRNA targeting sequences were distinct from the siRNA oligonucleotide targeting sequence thus serving as an additional control for the RNAi studies. Characterization of the shRNA cell lines revealed that downregulation of Endo180 expression had no impact on cell proliferation (Figure 6B). Using an assay of invasion into thick collagen gels, it was demonstrated that control shRNA glioma cells readily penetrate into the gel. In contrast, Endo180 shRNA cells showed a drastic impairment in their ability to invade through a fibrillar collagen I matrix (*p* = 0.0099) (Figure 6C).

## Discussion

The current study shows that Endo180 expression is negligible in the normal brain but highly upregulated in GBMs as revealed by expression array analysis and immunohistochemical staining of patient material. Endo180 is a well-characterized collagen internalization receptor and its extensive expression in GBMs required us to evaluate the collagen content in these tumors. Normal brain ECM is largely devoid of collagens, although some collagen IV can be detected in vascular basement membranes [5]. Masson's trichrome staining of a large panel of high-grade gliomas revealed that two types of collagen depositions were present in GBMs: the basement membrane collagens seen near microvascular proliferations, consisting mainly of collagen IV, and fibrillar collagens associated with adjacent stromal regions and present within the tumor mass. The existence of fibrillar collagen within the tumor mass is largely unappreciated in the literature as it was previously reported that the archetypical fibrillar collagen, collagen I, is absent in gliomas [8], [9], with rare exceptions as described for gliofibromas [33]. In contrast, our study shows that in a subset of GBMs, collagen I is an integral part of the ECM as revealed by collagen I antibody staining. This discrepancy between reports is most likely related to the detection method employed. When we repeated these previous studies and used peroxidase detection for collagen I immunohistochemical staining, we obtained an uninterpretable high background labeling. However, employing a recently optimized technique for high resolution multiple immunofluorescence labeling of FFPE tissue followed by confocal microscopy imaging [34] allowed us to reduce the background staining level and clearly visualize the collagen I deposition at higher magnification (Figure 5B). This deposition of intratumoral extracellular matrix components has three possible origins: they could be produced by adjacent stromal cells, by normal brain cells that are activated by the invading glioma cells [35] and/or by the tumor cells themselves as part of their mesenchymal differentiation (see below) [36]. Our studies demonstrate that tumor cells do indeed contribute to the production and deposition of collagen I (Figure 4D, Figure 5B). Importantly, the positive correlation between intratumoral collagen deposition and Endo180 expression observed in the high-grade glioma TMA means that Endo180 will be in intimate contact with is extracellular ligand and can thereby promote localized matrix remodeling and turnover.

High-grade gliomas (grade III and IV) have been classified into three subclasses on basis of their expression profiles: pro-neural, proliferative and mesenchymal [24]. Interrogation of these array data placed Endo180 firmly into the mesenchymal subclass, which is mainly associated with grade IV tumors, predicts for poor disease outcome and is characterized by excessive neovascularization. The association of Endo180 with the mesenchymal subclass is consistent with previous observations that Endo180 expression in normal tissue is predominantly restricted to cells of mesenchymal origin [13], [14], [15]. In tumors, a shift towards a more mesenchymal phenotype is a common feature of disease progression for which Endo180 could serve as a surrogate marker. Consistent with this, the expression of Endo180 in GBMs is correlated with tumor grade. Moreover, although tumors of epithelial origin are predominantly Endo180-negative, expression of Endo180 in 3–6% of invasive breast cancers correlated with a basal phenotype and shorter disease-free survival [37]. One of the potential triggers for a shift towards a more mesenchymal phenotype is TGF-β, a regulatory cytokine playing multiple roles in cancer [38] and hyperactivation of the downstream signaling pathway of TGF-β is often observed in poor prognosis gliomas [39]. Our *in silico* analysis of expression array data confirmed high expression of both TGF-β1 and TGF-β2 and its receptor TGF-β-RI in GBMs (Table S2). Interestingly, cell based studies demonstrated that TGF-β1 stimulation directly enhanced Endo180 expression in glioma cells (Figure 3), providing an explanation for the enhanced expression of Endo180 in GBMs.

Cell invasion *in vivo* requires that there is a balance between the requirement for a substrate to migrate on (usually composed of fibrillar matrix proteins) and the requirement for a space to migrate through. This study demonstrates, for the first time, that expression of Endo180 on the tumor cells is required for productive invasion into 3D collagen matrices (Figure 6). This result has two important implications. First, it provides a potential mechanism underlying the rapid disease progression displayed by GBMs compared to lower-grade astrocytic tumors. Second, it suggests that targeting Endo180 could limit the ECM turnover by tumor cells and thereby restrict GBM expansion. Together these data highlight how invading gliomas can balance the requirement for *de novo* matrix deposition and matrix remodeling and identifies a potential target for the treatment of this devastating disease.

## Materials and Methods

### Patient material

Brain tumor samples were obtained with written informed consent. The study, including the consent procedure, was conducted with specific approval from the Local and Multicentre Ethical Review Committees from the Royal Marsden NHS Foundation Trust, Sutton, St George's Hospital Medical School, London and King's College Hospital, London. Normal adult brain tissue from patients with no history of neurological disease were obtained from the UK Multiple Sclerosis Bank, Imperial College London. 11 cases were available on whole sections with an additional 79 cases present on a tissue microarray (TMA) with full clinicopathological data. The age of the patients ranged from 28–80 years (Table S1). All cases were reviewed by a pathologist (SP) to corroborate the diagnosis and assess the staining. Normal adult brain tissue from patients with no history of neurological disease were obtained from the UK Multiple Sclerosis Bank, Imperial College London [40].

### Antibodies and cells

Anti-Endo180 mAbs A5/158 and 39.10 directed against the extracellular domain of Endo180 have been previously described and characterized [11], [37]. Other antibodies were as follows: rabbit anti-glial fibrillary acidic protein (GFAP, Dako), rat anti-GFAP (Zymed Laboratories), rabbit anti-collagen I (BP8028, Acris), rabbit anti-collagen IV (AB6586, Acris), mouse anti-Smad2/3 (BD Biosciences), mAb phospho-Smad2 (Ser465/467, 138D4, Cell Signaling), anti-phospho-Smad3 (Ser423/425)/Smad1 (Ser463/465) (Cell Signaling), anti-vimentin (Dako), anti-α-tubulin antibody (Sigma), Alexa488 goat-anti-rabbit IgG, Alexa488 goat-anti-mouse IgG_2A_, Alexa555 goat-anti-mouse IgG_1_, and Alexa633 goat-anti-rat IgG (Invitrogen), HRP-conjugated goat-anti-mouse IgG (Jackson Immunoresearch), HRP-conjugated goat-anti-rabbit IgG (Santa Cruz) and APC-conjugated rat anti-mouse IgG_1_ (BD Biosciences). Human fibroblasts (HFFF2, human Caucasian fetal foreskin fibroblasts) were purchased from the European Collection of Cell Cultures (ECACC). All cell lines were maintained in DMEM with 10% FBS and 2.4 mM L-glutamine. For growth factor stimulation, cells were transferred into low serum medium (DMEM with 1% FBS) for 24 h before treatment for 24 or 72 h with 5 ng/ml recombinant human TGF-β1 (R&D systems), 50 ng/ml PDGF-BB (R&D Systems), or 50 ng/ml EGF (Invitrogen) in DMEM with 1% FBS.

### Immunostaining, Masson's trichrome staining and confocal imaging

For immunohistochemistry, 3 µm FFPE sections were dewaxed in xylene, rehydrated through a series of graded alcohols to water and subjected to high-temperature antigen retrieval in 0.01 M pH 6.0 citrate target retrieval buffer (Dako). Slides were allowed to cool for 20 min at room temperature and then incubated with anti-Endo180 mAb 39.10 (20 µg/ml) or 1∶1000 anti-GFAP for 1 h at room temperature. Detection was achieved with the Vectastain avidin-biotin complex (ABC) system according to the manufacturer's protocol (Vector Laboratories, Burlingame, CA, USA). Slides were rehydrated through a series of alcohols, cleared in xylene and mounted in 1,3-diethyl-8-phenylxanthine. Adjacent sections were stained with haematoxylin and eosin (H&E) or by the Masson's trichrome technique to visualize collagen fibers. Images were captured on a Leica DMRA2 microscope fitted with a Leica DFC320 camera.

A TMA consisting of 79 WHO grade III and IV gliomas was stained with the anti-Endo180 mAb 39.10 and the Masson's trichrome technique. Endo180 positivity was recorded for cores with widespread moderate to strong immunoreactivity, with samples containing only occasional weak staining tumor cells regarded as negative. Cores were considered positive for intratumoral collagen deposition where specific blue/green staining was observed within the tumor mass by the Masson's trichrome, and distinct from staining of the vascular basement membrane.

The protocol for the staining of FFPE material for confocal microscopy is described elsewhere [34], [41]. Fluorescent images were captured on a Leica Microsystems TCS-SP2 confocal.

### RNAi

The Endo180 targeting and Endo180 reversed siRNA oligonucleotides, and the protocol for cell transfection has been previously described [16]. For shRNA downregulation, cells were plated at 1.6×10^4^ cells per well in a 96-well plate overnight. The following day, the media was replaced with fresh media containing hexadimethrine bromide (8 µg/ml) (Sigma) and Mission lentiviral particles (Sigma) TRCN0000029674 (Endo180 shRNA) or TRCN0000068276 (control shRNA) added at a multiplicity of infection (MOI) of 10. Cells were incubated for 12 h before replacing the media with fresh DMEM plus 10% FBS. 36 h later, the medium was replaced with DMEM plus 10% FBS and puromycin (3 µg/ml) and cultured for a further 14 days. Cell populations were then cultured without puromycin.

### Reporter assays

Promoter fragments were generated by PCR with a reverse primer that started at −1 position of the ATG of Endo180 and four different forward primers located at different positions in the Endo180 promoter, generating products of 1498bp, 1145bp, 649bp and 390bp respectively. The sequence for reverse primer hEndo180R-0 bp was 5′-AAAAGCTTCCCCGAGCGCGGCGCTCAGT-3′ and for the four forward primers were: hEndo180F-1498bp: 5′-AAAGATCTAGCATTTCTGACCCAACACC-3′, hEndo180F-1146bp: 5′-TCTCAACGTCTCAGTCCTGC-3′, hEndo180F-649bp: 5′-GAGGTAGGGGATGCTAGGCT-3′, hEndo180F-390bp: 5′- GGGAGAGACTGGGAAACAGA-3′. The PCR products were cloned into the luciferase reporter vector, pGL3-basic (Promega) and verified by DNA sequencing. The −140bp/0bp Endo180 promoter fragment was generated by digestion of the pGL3 −1498bp/0bp vector with MscI and SmaI and self-ligating the digested vector. Smad binding elements in −1146bp/0bp Endo180 promoter fragment were mutated using the Quickchange II XL kit (Stratagene) and primers: mutSBE1F 5′-GTAACACTGAGTAACTGTGGATTAACTAACCTTTTATGAAATTTC-3′, mutSBE1R 5′-GAAATTTCATAAAAGGTTAGTTAATCCACAGTTACTCAGTGTTAC-3′, mutSBE2F 5′-GAGTGATGATGCCGAACTTCTGGGACGTCCAG-3′, mutSBE2R 5′-CTGGACGTCCCAGAAGTTCGGCATCATCACTC-3′. The pGL3 CAGA_12_-Luciferase reporter plasmid has been described previously [30]. The reporter assay was performed as previously described [42] with the following modifications. Briefly, U87MG cells were plated at low density. The next day, cells were transfected with the indicated reporter plasmids using FuGENE6 transfection reagent (Roche). The day after, cells were cultured in DMEM containing 1% FBS in the presence or absence of 5 ng/ml TGF-β1. After 24 h, cells were harvested in passive lysis buffer and luciferase activity was determined using the Promega luciferase assay system. In all transfections, a β-galactosidase expression plasmid (pDM2LacZ) was included to normalize the luciferase activities. β-galactosidase activity was determined in 100 mM Na_2_HPO_4_/NaH_2_PO_4_, 1 mM MgCl_2_, 100 mM 2-mercaptoethanol and 0.67 mg/ml O-nitrophenyl-galactopyranoside. Where indicated, cells were treated with the SMAD3 inhibitor, SIS3 (1.5 µM, Merck) [28] or with the TGF-β type I receptor inhibitor, SB431542 (20 µM, Tocris) [29], simultaneously with the TGF-β1 treatment.

### Collagen internalization

Cells were transfected with control or Endo180 siRNA oligonucleotides for 24 h and then treated with or without 5 ng/ml TGF-β1 for 72 h. Cells were then incubated with 20 µg/ml OG-collagen IV (Invitrogen) for 2 h in DMEM plus 1% FBS at 37°C before being harvested using trypsin/EDTA for 5 min at 37°C. Cells were stained on ice with anti-Endo180 mAb A5/158 or mouse IgG_1_ for 30 min followed by an APC-conjugated rat anti-mouse IgG_1_ antibody for 30 min. Cells were washed twice and FACS analyzed in the presence of propidium iodide.

### Invasion assays

For Transwell migration assays, siRNA transfected cells cultured for 24 h in DMEM with 1% FBS were trypsinized and 5×10^4^ cells were plated, in the same medium, onto 6.5 mm Transwell inserts (8.0 µm pores; Costar) that had been pre-coated with 50 µg/ml collagen I (PureCol, Inamed Biomaterials). The lower well contained DMEM plus 10% FBS. After 24 h, invaded cells on the underside of the filter were methanol fixed and stained with 1% toluidine blue/1% borax solution. The membrane was excised and three photos were taken of each insert at 10× magnification. Cells were counted using the ImageJ software.

Collagen gel invasion assays were performed as previously [43] with the following modifications. 5×10^5^ glioma cells were plated into 24 well plates containing a 1 ml gel comprising a 1∶1 mix of rat tail collagen type 1 (BD Biosciences; 3.7 mg/ml) and Matrigel (BD Biosciences; 12.1 mg/ml) and incubated overnight at 37°C. The following day, the cultures were raised to an air-liquid interface and fed from the underside every two days with DMEM plus 10% FBS and 50 ng/ml EGF (Invitrogen) for 11 days. At the end of the assay, gels were fixed in 4% paraformaldehyde before being formalin-fixed and embedded in paraffin wax. 3 µm sections were taken from the centre of each gel and immunostained with anti-vimentin antibody to detect the glioma cells. Images were analyzed using ImageJ software to count the number of invaded particles containing ≥1 cell (N), the total area invaded (A), and the depth of invasion (D). The invasion index is calculated as N×A×D [43].

### Bioinformatic analysis

A profile search for glioblastoma was performed on the ONCOMINE™ database [44]. Five expression profiling studies were selected that compared grade III gliomas (astrocytoma, oligodendroglioma and oligoastrocytoma) with grade IV gliomas (GBM) [19], [20], [21], [22], [23]. A ranked list of genes differentially expressed between grade III and IV tumors across all of these studies was calculated by T statistics. For the expression data from Phillips and colleagues [24] differential expression between the classes was calculated for the two *MRC2* probe sets using the Mann-Whitney U test.

## Supporting Information

Table S1 (0.08 MB PDF)

Table S2 (0.05 MB PDF)

Figure S1 Endo180 promoter methylation. Methylation of Endo180 in intronic region +375bp/+579bp was monitored using a methylation-sensitive PCR (MSP). Briefly, 1 µg of genomic DNA from normal brain and 19 glioma cell lines was subjected to sodium bisulphite conversion using EZ DNA Methylation Kit (Zymo Research) after which MSP was performed in a reaction volume of 20 µl for 40 cycles. Amplification products were resolved on 1.5% agarose gels and visualized under UV illumination to compare unmethylated (U) and methylated (M) amplifications. The following Endo180 primers were used: MRC2U-F 5′-ATTTTAGTAGTTTAGGAGGAAGTGG-3′. MRC2U-R 5′-TAATTAAAAAACCATCCTAACACA-3′. MRC2M-F 5′-GGATTTTAGTAGTTTAGGAGGAAGC-3′. MRC2M-R 5′-AATAATTAAAAAACCGTCCTAACG-3′. In 18 out of 19 cell lines and in normal brain no promoter methylation was detected. Cell line UW479 showed a methylated promoter status that corresponded with an absence of Endo180 gene transcript as tested by qPCR (see Figure 4C).
(0.29 MB TIF)
