# Supplementary material for: A Role for Fibrillar Collagen Deposition and the Collagen Internalization Receptor Endo180 in Glioma Invasion
Source: PLoS One. 2010 Mar 22;5(3):e9808. doi: 10.1371/journal.pone.0009808 (PMC2842440; doi:10.1371/journal.pone.0009808)
Supplement: Table S1 — (0.08 MB PDF) [file pone.0009808.s001.pdf]

Table S1: List of 100 most upregulated genes in GBM versus grade III gliomas.

Rank	Gene symbol	Gene name	Up in GBM (p-value)
1	NNMT	Nicotinamide n-methyltransferase	1.80×10^{-6}
2	TncRNA	Throphoblast-derived noncoding RNA	2.22×10^{-6}
3	PDPN	Podoplanin	9.31×10^{-6}
4	IGFBP5	Insulin-like growth factor binding protein 5	1.96×10^{-5}
5	SOD2	Superoxide dismutase 2	3.03×10^{-5}
6	MRC2/Endo180	Mannose receptor, C-type 2; Endo180	5.56×10^{-5}
7	SHC1	Shc transforming protein	5.72×10^{-5}
8	PLAT	Plasminogen activator, tissue	1.12×10^{-4}
9	ITGA7	Integrin, alpha 7	1.42×10^{-4}
10	CD151	CD151	1.71×10^{-4}
11	CALD1	Caldesmon 1	1.88×10^{-4}
12	VEGFA	Vascular Endothelial Growth Factor A	2.21×10^{-4}
13	DDOST	Dolichyl-diphosphooligosaccharide-protein glycosyltransferase	2.34×10^{-4}
14	SERPINE1	Serpin peptidase inhibitor, clade E; PAI-1	3.02×10^{-4}
15	ABCC3	ATP-binding cassette, sub-family C (CFTR/MRP), member 3	3.14×10^{-4}
16	ADM	Adrenomedullin	4.33×10^{-4}
17	COL5A2	Collagen, type V, alpha2	5.66×10^{-4}
18	DNALI1	Dynein, axonemal, light intermediate chain 1	5.76×10^{-4}
19	PRSS23	Protease, serine, 23	6.89×10^{-4}
20	CSDA	Cold shock domain protein A	7.04×10^{-4}
21	SP100	SP100 nuclear antigen	7.13×10^{-4}
22	CAV1	Caveolin 1, caveolae protein, 22kDa	7.77×10^{-4}
23	GDF15	Growth Differentiation Factor 15	8.49×10^{-4}
24	ITGA3	Integrin, alpha 3	9.82×10^{-4}
25	TM9SF1	Transmembrane 9 superfamily member 1	9.94×10^{-4}
26	MEOX2	Mesenchyme homeobox 2	0.001
27	PROS1	Protein S (alpha)	0.001
28	TGFB2	Transforming growth factor, beta 2	0.001
29	P4HB	Proline 4-hydroxylase, beta	0.001
30	THBS1	Thrombospondin 1	0.001
31	COL1A2	Collagen, type I, alpha2	0.002
32	CD44	CD44	0.002
33	FGFR1	Fibroblast Growth Factor Receptor 1	0.002
34	PHC2	Polyhomeotic homolog 2 (Drosophila)	0.002
35	H2AFV	H2A histone family, member V	0.002
36	PDIA5	Protein disulfide isomerase family A, member 5	0.002
37	TAGLN	Transgelin	0.002
38	RGS6	Regulator of G-protein signaling 6	0.003
39	TRIP6	Thyroid hormone receptor interactor 6	0.003
40	MPZL2	Myelin protein zero-like 2	0.003
41	IL1RAP	Interleukin 1 receptor accessory protein	0.003
42	C1R	Complement component 1, r subcomponent	0.003
43	COL6A1	Collagen, type VI, alpha1	0.003
44	CA12	Carbonic Anhydrase XII	0.003
45	SFRP4	Secreted frizzled-related protein 4	0.003
46	MCM4	Minichromosome maintenance complex component 4	0.003
47	DDB2	Damage-specific DNA binding protein 2, 48kDa	0.003
48	FKBP9	FK506 binding protein 9, 63 kDa	0.003
49	FN1	Fibronectin 1	0.003
50	GNS	Glucosamine (N-acetyl)-6-sulfatase (Sanfilippo disease IIID)	0.003
51	UPP1	Uridine phosphorylase 1	0.003
52	CRIP1	Cysteine-rich protein 1 (intestinal)	0.004
53	PLOD3	Procollagen-lysine, 2-oxoglutarate 5-dioxygenase 3	0.004
54	CDKN1A	Cyclin-Dependent Kinase Inhibitor 1A (p21, Cip1)	0.004
55	LAMA4	Laminin, alpha 4	0.004
56	BST2	Bone marrow stromal cell antigen 2	0.004
57	GUSB	Glucuronidase, beta	0.004
58	ELF4	E74-like factor 4 (ets domain transcription factor)	0.004
59	HLA-DQB1	Major histocompatibility complex, class II, DQ beta 1	0.004

60	EMP3	Epithelial membrane protein 3	0.004
61	COL4A1	Collagen, type IV, alpha 1	0.004
62	EYA2	Eyes absent homolog 2 (Drosophila)	0.004
63	MMP9	Matrix metallopeptidase 9	0.004
64	HSPG2	Heparan sulfate proteoglycan 2	0.005
65	LIMS1	LIM and senescent cell antigen-like domains 1	0.005
66	COL4A2	Collagen, type IV, alpha 2	0.005
67	MOXD1	Monooxygenase, DBH-like 1	0.005
68	TGFB1	Transforming growth factor, beta-induced	0.006
69	TGFBR1	Transforming growth factor, beta receptor I	0.006
70	TAP1	Transporter 1, ATP-binding cassette, sub-family B (MDR/TAP)	0.006
71	HIST1H2BD	Histone cluster 1, H2bd	0.006
72	SLC22A18	Solute carrier family 22 (organic cation transporter), member 18	0.006
73	CDC25B	Cell division cycle 25 homolog B (S. pombe)	0.006
74	MR1	Major histocompatibility complex, class I-related	0.006
75	KDELR1	KDEL (Lys-Asp-Glu-Leu) endoplasmic reticulum protein retention receptor 1	0.006
76	TSPAN4	Tetraspanin 4	0.006
77	CFLAR	CASP8 and FADD-like apoptosis regulator	0.006
78	RARRES2	Retinoic acid receptor responder (tazarotene induced) 2	0.006
79	GPX7	Glutathione peroxidase 7	0.006
80	FAM114A1	Family with sequence similarity 114, member A1	0.007
81	PLA2G2A	Phospholipase A2, group IIA (platelets, synovial fluid)	0.007
82	MXRA5	Matrix-remodelling associated 5	0.007
83	GLB1	Galactosidase, beta 1	0.007
84	RBPMS	RNA binding protein with multiple splicing	0.007
85	DIRAS3	DIRAS family, GTP-binding RAS-like 3	0.007
86	ANGPT2	Angiopoietin 2	0.007
87	RAB27A	RAB27A, member RAS oncogene family	0.008
88	CCR1	Chemokine (C-C motif) receptor 1	0.008
89	HEXA	Hexosaminidase A (alpha polypeptide)	0.008
90	BGN	Biglycan	0.008
91	RUNX1	Runt-related transcription factor 1 (acute myeloid leukemia 1; aml1 oncogene)	0.008
92	ARL4C	ADP-ribosylation factor-like 4C	0.008
93	FEM1C	Fem-1 homolog c (C. elegans)	0.008
94	STAT1	Signal transducer and activator of transcription 1, 91kDa	0.008
95	WEE1	WEE1 homolog (S. pombe)	0.009
96	OAS2	2'-5'-oligoadenylate synthetase 2, 69/71kDa	0.009
97	HLA-A	Major histocompatibility complex, class I, A	0.009
98	TRADD	TNFRSF1A-associated via death domain	0.009
99	KLF10	Kruppel-like factor 10	0.009
100	DPYD	Dihydropyrimidine dehydrogenase	0.010

An ONCOMINE™ profile search for glioblastoma comparing 5 independent studies; Shai_brain, Liang_brain, Sun_brain and Bredel_brain_2 and Freye_brain was undertaken (see Figure 1A). A ranked list of genes differentially expressed between grade III and IV tumors across all of these studies was compiled and *p*-values calculated by T statistics. Highlighted are Endo180 (*MRC2*, green), genes involved in TGF-β signaling (orange) and ECM proteins, ECM receptors and ECM proteases (yellow).
