# Supplementary material for: A Role for Fibrillar Collagen Deposition and the Collagen Internalization Receptor Endo180 in Glioma Invasion
Source: PLoS One. 2010 Mar 22;5(3):e9808. doi: 10.1371/journal.pone.0009808 (PMC2842440; doi:10.1371/journal.pone.0009808)
Supplement: Table S2 — (0.05 MB PDF) [file pone.0009808.s002.pdf]

Table S2. Clinicopathological details, Endo180 expression and intratumoral collagen deposition in 79 high-grade gliomas

Case no.	Diagnosis	WHO grade	Sex	Age (years)	Endo180	Intratumoural Collagen
**1**	Anaplastic oligodendroglioma	III	Male	57	Negative	Negative
**2**	Anaplastic oligodendroglioma	III	Female	63	Negative	Negative
**3**	Anaplastic oligodendroglioma	III	Female	46	Negative	Negative
**4**	Anaplastic oligodendroglioma	III	Male	36	Positive	Negative
**5**	Anaplastic oligodendroglioma	III	Male	28	Positive	Negative
**6**	Anaplastic astrocytoma	III	Male	65	Positive	Positive
**7**	Anaplastic astrocytoma	III	Male	38	Negative	Negative
**8**	Anaplastic astrocytoma	III	Female	75	Negative	Negative
**9**	Anaplastic astrocytoma	III	Female	38	Negative	Negative
**10**	Anaplastic astrocytoma	III	Male	52	Negative	Negative
**11**	Glioblastoma multiforme	IV	Male	42	Positive	Negative
**12**	Glioblastoma multiforme	IV	Male	76	Positive	Negative
**13**	Glioblastoma multiforme	IV	Female	63	Negative	Negative
**14**	Glioblastoma multiforme	IV	Male	70	Positive	Negative
**15**	Glioblastoma multiforme	IV	Female	56	Positive	Positive
**16**	Glioblastoma multiforme	IV	Male	50	Positive	Negative
**17**	Glioblastoma multiforme	IV	Male	53	Positive	Positive
**18**	Glioblastoma multiforme	IV	Female	57	Positive	Negative
**19**	Glioblastoma multiforme	IV	Male	76	Positive	Negative
**20**	Glioblastoma multiforme	IV	Female	67	Negative	Negative
**21**	Glioblastoma multiforme	IV	Male	55	Positive	Positive
**22**	Glioblastoma multiforme	IV	Male	42	Positive	Negative
**23**	Glioblastoma multiforme	IV	Male	60	Positive	Positive
**24**	Glioblastoma multiforme	IV	Male	40	Positive	Negative
**25**	Glioblastoma multiforme	IV	Male	47	Negative	Negative
**26**	Glioblastoma multiforme	IV	Male	41	Positive	Positive
**27**	Glioblastoma multiforme	IV	Female	74	Positive	Negative
**28**	Glioblastoma multiforme	IV	Male	70	Positive	Negative
**29**	Glioblastoma multiforme	IV	Female	44	Negative	Negative
**30**	Glioblastoma multiforme	IV	Female	67	Positive	Negative
**31**	Glioblastoma multiforme	IV	Female	61	Positive	Negative
**32**	Glioblastoma multiforme	IV	Male	72	Positive	Negative
**33**	Glioblastoma multiforme	IV	Female	79	Positive	Positive
**34**	Glioblastoma multiforme	IV	Male	56	Positive	Negative
**35**	Glioblastoma multiforme	IV	Male	65	Positive	Positive
**36**	Glioblastoma multiforme	IV	Male	52	Positive	Positive
**37**	Glioblastoma multiforme	IV	Male	51	Negative	Negative
**38**	Glioblastoma multiforme	IV	Female	55	Positive	Negative
**39**	Glioblastoma multiforme	IV	Male	66	Negative	Negative
**40**	Glioblastoma multiforme	IV	Female	53	Positive	Negative
**41**	Glioblastoma multiforme	IV	Female	54	Positive	Negative

**42**	Glioblastoma multiforme	IV	Male	61	Positive	Positive
**43**	Glioblastoma multiforme	IV	Male	80	Positive	Negative
**44**	Glioblastoma multiforme	IV	Male	50	Positive	Positive
**45**	Glioblastoma multiforme	IV	Male	39	Positive	Negative
**46**	Glioblastoma multiforme	IV	Male	68	Positive	Negative
**47**	Glioblastoma multiforme	IV	Male	59	Positive	Negative
**48**	Glioblastoma multiforme	IV	Female	44	Positive	Negative
**49**	Glioblastoma multiforme	IV	Female	60	Positive	Negative
**50**	Glioblastoma multiforme	IV	Male	63	Positive	Negative
**51**	Glioblastoma multiforme	IV	Male	73	Positive	Negative
**52**	Glioblastoma multiforme	IV	Female	59	Positive	Negative
**53**	Glioblastoma multiforme	IV	Male	58	Positive	Positive
**54**	Glioblastoma multiforme	IV	Male	47	Negative	Negative
**55**	Glioblastoma multiforme	IV	Male	57	Positive	Negative
**56**	Glioblastoma multiforme	IV	Male	48	Positive	Negative
**57**	Glioblastoma multiforme	IV	Male	75	Positive	Positive
**58**	Glioblastoma multiforme	IV	Male	49	Negative	Negative
**59**	Glioblastoma multiforme	IV	Female	34	Positive	Negative
**60**	Glioblastoma multiforme	IV	Female	69	Positive	Positive
**61**	Glioblastoma multiforme	IV	Male	69	Positive	Positive
**62**	Glioblastoma multiforme	IV	Male	69	Negative	Negative
**63**	Glioblastoma multiforme	IV	Female	53	Positive	Negative
**64**	Glioblastoma multiforme	IV	Female	66	Positive	Negative
**65**	Glioblastoma multiforme	IV	Male	78	Positive	Negative
**66**	Glioblastoma multiforme	IV	Male	63	Positive	Positive
**67**	Glioblastoma multiforme	IV	Male	63	Positive	Negative
**68**	Glioblastoma multiforme	IV	Male	67	Positive	Negative
**69**	Glioblastoma multiforme	IV	Male	71	Positive	Negative
**70**	Glioblastoma multiforme	IV	Female	68	Positive	Positive
**71**	Glioblastoma multiforme	IV	Female	80	Positive	Positive
**72**	Glioblastoma multiforme	IV	Female	64	Positive	Negative
**73**	Glioblastoma multiforme	IV	Female	38	Negative	Negative
**74**	Glioblastoma multiforme	IV	Male	66	Positive	Positive
**75**	Glioblastoma multiforme	IV	Female	68	Positive	Negative
**76**	Glioblastoma multiforme	IV	Female	68	Positive	Positive
**77**	Glioblastoma multiforme	IV	Female	63	Positive	Positive
**78**	Glioblastoma multiforme	IV	Male	71	Positive	Negative
**79**	Glioblastoma multiforme	IV	Female	38	Positive	Positive

Endo180 immunohistochemical staining and Masson's trichrome staining to detect fibrillar collagens was as described in Figure 5. Statistical data is shown in Table 1.
