# Supplementary figures and images for: A Role for Fibrillar Collagen Deposition and the Collagen Internalization Receptor Endo180 in Glioma Invasion
Source: PLoS One. 2010 Mar 22;5(3):e9808. doi: 10.1371/journal.pone.0009808 (PMC2842440; doi:10.1371/journal.pone.0009808)

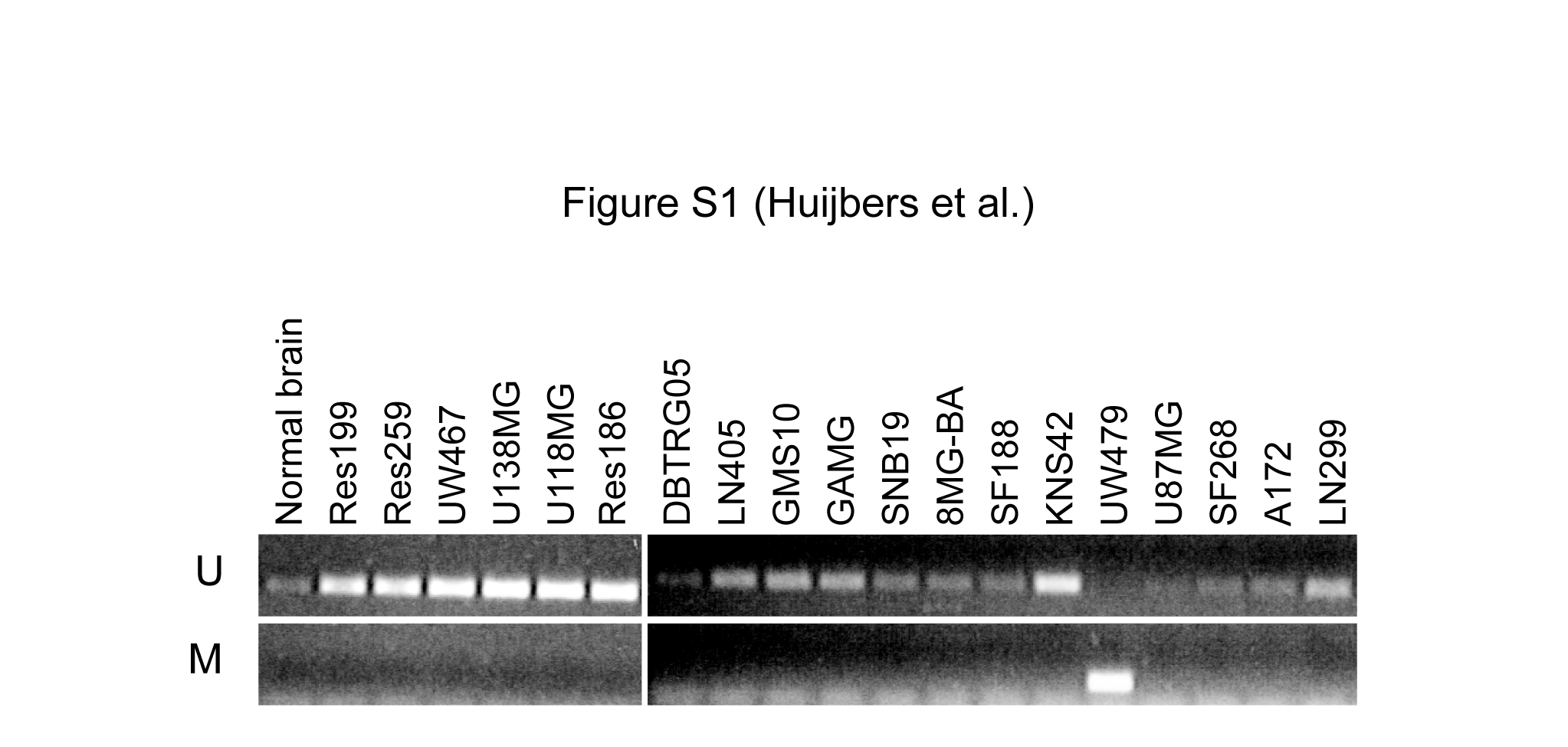

Supplement: Figure S1 — Endo180 promoter methylation. Methylation of Endo180 in intronic region +375bp/+579bp was monitored using a methylation-sensitive PCR (MSP). Briefly, 1 µg of genomic DNA from normal brain and 19 glioma cell lines was subjected to sodium bisulphite conversion using EZ DNA Methylation Kit (Zymo Research) after which MSP was performed in a reaction volume of 20 µl for 40 cycles. Amplification products were resolved on 1.5% agarose gels and visualized under UV illumination to compare unmethylated (U) and methylated (M) amplifications. The following Endo180 primers were used: MRC2U-F 5′-ATTTTAGTAGTTTAGGAGGAAGTGG-3′. MRC2U-R 5′-TAATTAAAAAACCATCCTAACACA-3′. MRC2M-F 5′-GGATTTTAGTAGTTTAGGAGGAAGC-3′. MRC2M-R 5′-AATAATTAAAAAACCGTCCTAACG-3′. In 18 out of 19 cell lines and in normal brain no promoter methylation was detected. Cell line UW479 showed a methylated promoter status that corresponded with an absence of Endo180 gene transcript as tested by qPCR (see Figure 4C). (0.29 MB TIF) [file pone.0009808.s003.tif]
